# Mimicking Myelodysplastic Syndrome: Importance of Differential Diagnosis

**DOI:** 10.1155/2021/9661765

**Published:** 2021-11-29

**Authors:** Thomas Luo, Joanna Zurko, John Astle, Nirav N. Shah

**Affiliations:** ^1^BMT & Cellular Therapy Program, Division of Hematology & Oncology, Medical College of Wisconsin, Milwaukee, WI, USA; ^2^Department of Pathology, Medical College of Wisconsin, Milwaukee, WI, USA

## Abstract

Copper deficiency is a rare nutritional deficiency with hematological manifestations that mimic those found in myelodysplastic syndrome, a hematological malignancy incurable without allogeneic hematopoietic stem cell transplantation. Bone marrow biopsy findings and peripheral blood counts are oftentimes insufficient to differentiate the two conditions. Moreover, the symptoms of copper deficiency can arise years after the surgery, making diagnosis a challenge. In patients with new-onset pancytopenia, copper deficiency must be considered on the differential, especially in the setting of known risk factors such as bariatric surgery, zinc supplementation, and celiac disease. Herein, we present a case of a 61-year-old female with a remote history of gastric bypass being evaluated for MDS in the context of progressive pancytopenia and new-onset paresthesias. The patient was found to have low serum copper and ceruloplasmin. Copper supplementation largely resolved the hematological abnormalities, but the limb paresthesias remain. This case highlights the need to identify copper deficiency early and distinguish it from MDS in order to prevent permanent neurological deficits and catastrophic response should the patient undergo hematopoietic stem cell transplantation.

## 1. Introduction

Myelodysplastic syndrome (MDS) is a hematological malignancy that presents with pancytopenia, ineffective hematopoiesis, and cytogenetic abnormalities; the condition is considered incurable without an allogeneic stem cell transplant (allo-HCT) [1]. The diagnosis is made through bone marrow biopsy evaluation and requires appropriate exclusion of other causes of cytopenias, e.g., nutritional deficiencies, drug toxicities, infection, and autoimmune disease [2]. Several conditions can mimic MDS with potentially catastrophic consequences should the patient proceed towards allo-HCT [3]. In this report, we describe a case of copper deficiency initially diagnosed as MDS to emphasize the need to rule out nutritional deficiency in the workup of patients with suspected MDS. IRB submission was deferred per institutional policy for case series including less than 3 patients.

## 2. Case Report

A 61-year-old female with a history of alcoholism, vitamin B12 deficiency, and remote gastric bypass was referred to hematology with a two-month history of shortness of breath and cytopenias. She admitted to consuming 2–4 alcoholic drinks weekly and had just recently resumed B12 supplementation. Complete blood count revealed a severe macrocytic anemia (6.6 g/dL), MCV (108 fl), and neutropenia (ANC 520/*μ*L). She had an elevated vitamin B12 level reflective of recent intramuscular supplementation (Table 1). Bone marrow biopsy was performed and revealed a hypercellular marrow for age (75%) and dyserythropoiesis but without cytogenetic and dysplastic changes characteristic of MDS. However, early MDS could not be ruled out. Despite two months of B12 supplementation, her anemia and leukopenia did not improve, and she required further transfusion. Direct Coombs testing, lactate dehydrogenase, reticulocyte count, haptoglobin, and methylmalonic acid testing revealed no significant abnormalities. Daily prednisone (40 mg) was started as empiric treatment for potential occult autoimmune condition with no efficacy. The patient's anemia continued to worsen (5.7 g/dL) requiring further transfusion with progressive neutropenia. Additionally, she developed bilateral foot paresthesias, which were felt to be due to Raynaud's phenomena. A repeat bone marrow biopsy now revealed a hypocellular bone marrow (20–30%) with erythroid hyperplasia and dyserythropoiesis. A presumptive diagnosis of MDS (R–IPSS 3) was made, and the patient was referred for allo-HCT evaluation.

**Figure 1:**
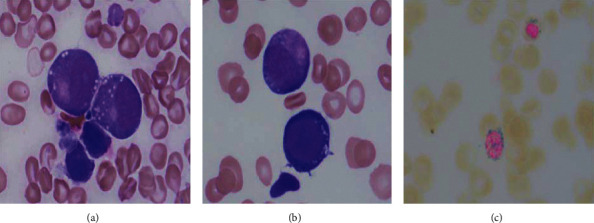
(a-b) Wright Giemsa-stained bone marrow aspirate smear slide (l000x magnification) showing neutrophil precursors (a) and an erythroid precursor (b) bottom with cytoplasmic vacuoles. (c) Prussian blue-stained bone marrow aspirate smear slide (l000x magnification) showing ring sideroblasts.

At time of consultation, the patient reported taking a daily zinc supplement for several years for a previously diagnosed deficiency. The combined history of gastric bypass and high zinc intake raised suspicion for copper deficiency, and additional labs were drawn. Both serum ceruloplasmin (<3 mg/dL) and copper (10 *μ*g/dL) were below the lower limits of diagnostic testing, and serum zinc (151 *μ*g/dL) was elevated. Hematopathology review of the second bone marrow biopsy showed vacuolated precursors and ring sideroblasts commonly seen in copper deficiency (Figure 1). Accordingly, the patient was started on IV cupric chloride (2 g) daily for 5 days and then once weekly for four weeks. At one-month follow-up, the patient's anemia improved and leukopenia resolved (Table 1). Although her energy level improved, her paresthesia remained.

## 3. Discussion

Copper deficiency is a rare nutritional condition that results in reversible hematologic findings—anemia and neutropenia—and often irreversible neurologic manifestations—optic neuropathy, paresthesia, and spasticity [4]. The most common etiologies of copper deficiency in the United States are gastric bypass surgery, excessive zinc intake, and celiac disease [4, 5]. Since the majority of copper is absorbed in the stomach and duodenum, gastric bypass surgery significantly increases risk of copper deficiency [4, 6]. The utility of prophylactic oral copper supplementation or serum copper monitoring in bariatric surgery patients is well established [6, 7]. Zinc primarily upregulates expression of metallothionein in enterocytes, which binds copper to a greater extent than zinc, thereby sequestering copper within the intestinal tract [4, 8].

While our patient had risk factors for development of copper deficiency, her age and clinical presentation were suggestive of MDS [9]. The early signs of insufficiency of hematopoiesis seen in copper deficiency—cytopenias and dysplastic changes—mimic those seen in clonal expansion driven diseases such as MDS—a condition encountered more frequently [10, 11]. Although not pathognomonic for copper deficiency, intracytoplasmic vacuoles in precursor cells and ring sideroblasts are subtle findings often observed on bone marrow biopsy as found in this patient; cytogenetic changes are not found in copper deficiency [10, 12]. Although the mechanism of copper deficiency anemia is unclear, copper is a vital coenzyme in the function of hephaestin—responsible for the oxidation of dietary iron—and ceruloplasmin—responsible for the transfer of iron from macrophages/monocytes to plasma [4, 13].

## 4. Conclusion

In this report, we highlight the clinical progression of occult copper deficiency. Although our patient's copper deficiency was initially misdiagnosed, prompt treatment with IV cupric chloride ameliorated the hematological and constitutional symptoms of low copper. Since delayed treatment of copper deficiency can result in permanent neurological impairment, clinicians should promptly rule out copper deficiency in patients being evaluated for MDS, especially if presenting with neurological symptoms [4, 14]. The steady rise of bariatric surgeries performed to curb the obesity epidemic will likely increase the prevalence of nutritional deficiencies, including copper deficiency [15]. Most importantly, early diagnosis can alleviate symptoms and spare patients' toxicities associated with chemotherapeutic agents or allo-HCT.

## Figures and Tables

**Table 1:** Laboratory values.

	Initial presentation	+2 months	+4 months allo-HCT consult	1 month postcopper replacement
WBC, K/*µ*L	**1.3**	**1.4**	**0.8**	4.3
ANC, K/*µ*L	**0.7**	**0.4**	**0.29**	3.3
MCV	**108**	96	97	93
Hgb, g/dL	**6.6**	**7.1**	**7.3**	**11.6**
PLT, K/*µ*L	222	191	**144**	157
Vit B12 (211–911)	>2000	—	507	—
Copper (80–155)	—	—	**<10**	107
Ceruloplasmin (17–54)	—	—	**<3**	—

WBC, white blood cell; ANC, absolute neutrophil count; MCV, mean corpuscular volume; Hgb, hemoglobin; PLT, platelet; Vit, vitamin. Bolded values are abnormal.

## Data Availability

The data used to support the findings of this study are available from the corresponding author upon request.
